# Association of endothelial nitric oxide synthase (eNOS) gene polymorphisms and physical fitness levels with plasma nitrite concentrations and arterial blood pressure values in older adults

**DOI:** 10.1371/journal.pone.0206254

**Published:** 2018-10-18

**Authors:** Roberta Fernanda da Silva, Átila Alexandre Trapé, Thaís Amanda Reia, Riccardo Lacchini, Gustavo Henrique Oliveira-Paula, Lucas Cezar Pinheiro, José Eduardo Tanus-Santos, André Mourão Jacomini, Carlos Roberto Bueno Júnior, Anderson Saranz Zago

**Affiliations:** 1 Department of Physical Education, São Paulo State University (UNESP), School of Science, Bauru, SP, Brazil; 2 Ribeirão Preto College of Nursing, USP—University of São Paulo, Ribeirão Preto, SP, Brazil; 3 Faculty of Medicine of Ribeirão Preto, USP—University of São Paulo, Ribeirão Preto, SP, Brazil; Augusta University, UNITED STATES

## Abstract

Endothelial nitric oxide synthase (eNOS) gene polymorphisms are associated with reduced eNOS activity and nitric oxide (NO) production leading to an increase in blood pressure (BP). Regular exercise is the main strategy to minimize the deleterious effects of polymorphisms. However, due to the differences that physical exercise can be performed, some controversial results are found. Therefore it seems reasonable to evaluate the training status (TS). Thus, this study aimed to investigate the association of eNOS gene haplotypes and different levels of TS on nitrite concentrations (NO2-) and BP values in older adult. 424 elderly performed the following assessments: General Functional Fitness Index (GFFI) to estimate TS, systolic and diastolic blood pressure (SBP and DBP), blood collection for analysis of NO2- and g.-786T>C, intron 4b/a (VNTR) and 894G>T polymorphisms. Multivariate logistic regression showed that NO2- was influenced by GFFI and 4b/4a Intron 4. Regarding BP, GFFI influenced SBP and DBP, and just intron 4 was associated with variations in DBP. It can be observed that GFFI affected the NO2-, SBP and DBP independently of haplotypes. Therefore, maintenance of good level of TS can overcome the negative influence of genetics factors (intron 4) by increasing NO2- concentration and decreasing BP values.

## Introduction

Life expectancy as well as health problems have been increasing worldwide, especially cardiovascular diseases [1]. Hypertension (HT) is one of the most prevalent risk factors for cardiovascular diseases [1, 2] and its etiology is multifactorial, including genetic factors. Evidence supports the theory that several polymorphisms contribute to variations in the concentrations of some substances which influence blood pressure (BP) values. In this regard, the polymorphisms of endothelial nitric oxide synthase (*eNOS*) can influence nitric oxide (NO) formation, a powerful vasodilator with a critical role in BP control [3, 4]. Since *eNOS* is related to an important pathway of cardiovascular control, the presence of variant allele polymorphisms in the gene that codes for *eNOS* could interfere directly in BP values [5].

The most studied variations of the *eNOS* gene (g.-786T>C (promoter); 894G>T (exon7); and, intron 4b/a (VNTR)) are associated with reduced *eNOS* activity and reduced NO production. As a consequence, there is an increase in vascular resistance and BP values [6, 7]. In addition, these effects seem to be increased during the aging process [8, 9].

Conversely, regular practice of physical exercise is considered as a non-pharmacological therapeutic tool to attenuate these effects. People who practice physical exercise present lower BP values ​​compared with sedentary people [10]. In addition, studies report that regular exercise promotes some benefits that minimize the deleterious effects of such polymorphisms. The main mechanism related to this is shear stress, which stimulates NO production and vasodilatation, contributing to a reduction on BP values [11].

However, the overall effectiveness of physical exercise on BP and NO production is still controversial, in part, due to the difference in type, intensity, and duration of exercise program interventions [12–14]. Therefore, it seems reasonable to evaluate the training status (TS), not only because it provides general assessment of physical fitness, but also it takes into account all types of physical exercise that can be performed.

The literature lacks a direct evaluation of eNOS polymorphisms with changes in NO production and BP values taking into account the TS as a confounding factor. Actually in a recent publication of our group [3], it was demonstrated that the maintenance of good levels of TS, particularly in individuals who are C allele carriers for the g.-786T>C polymorphism combined with T allele carriers for the 894G>T polymorphism, resulted in an increase in nitrite plasma concentrations (NO_2_^-^), which may reflect in improved NO bioavailability. However this study was developed in normotensive older adult participants and further studies still need to be performed considering hypertensive and normotensive individuals together.

Thus, the purpose of the current study was to investigate the association of *eNOS* gene polymorphisms and different levels of TS on NO production and BP values in older adults. The hypotheses tested in this study were: (a) *eNOS* gene polymorphism and TS level are independently associated with changes in NO production and BP values in older adults, (b) variants of *eNOS* gene polymorphisms are associated with reduced NO production and increased BP values in older adults, and (c) good levels of TS can make carriers of detrimental *eNOS* polymorphisms reach the same NO and BP levels as ancestral allele carriers.

## Materials and methods

### Screening

Individuals from extension programs and retired community associations linked to the São Paulo State University (UNESP)—Bauru/ SP/ Brazil and the University of São Paulo (USP)–Ribeirão Preto/SP/Brazil were invited to participate in this study. In total, 424 subjects who met the following inclusion criteria participated in the present study: non-smoking; non-alcoholics (<3 drinks per day); aged between 40 and 80 years; non-diabetic (fasting glucose level lower than 100 mg/dL); not having cardiovascular (angina, peripheral or cerebrovascular disease, etc.), neurological, or psychiatric diseases; and not presenting other known medical or orthopedic conditions that prevent participation in physical tests.

The study was approved by the Institutional Review Board of São Paulo State University—UNESP (CEP/FC-UNESP n^o^ 323.427) and the Institutional Review Board of University of São Paulo—USP (CEP/FCFRP n^o^.172). All subjects provided their written informed consent prior to beginning the experiments.

### Clinical assessment

BP was measured after 5 minutes of rest on three separate days according to the VII Brazilian Hypertension Guidelines [15], using an aneroid sphygmomanometer (Wan Med) adapted to the circumference of the arm and a stethoscope (Littmann) placed over the brachial artery. The body mass index (BMI) was calculated as the ratio of weight to the square of height (kg/m^2^), as described by Pollock and Wilmore [16].

### Blood measurements

Blood samples were collected in the morning, two hours after breakfast and participants were instructed to avoid foods with a high concentration of nitrate on the day before collection, such as beetroot, arugula, spinach, lettuce, and others. Forearm venous blood samples were collected in standard Vacutainer tubes (Becton–Dickinson, Brazil) containing EDTA or heparin. Tubes were immediately centrifuged at 2,000g for 5 minutes at room temperature, and plasma aliquots were stored at -80°C until assayed [17]. Plasma samples were used to evaluate the nitrite concentration (NO_2_^-^), a sensitive marker of NO production, using an ozone-based reductive chemiluminescence assay. For this, 50 μL of plasma samples was injected into a solution of acidified triiodide, purged with nitrogen in line with a gas-phase chemiluminescence NO analyzer (Sievers Model 280 NO Analyzer, Sievers, Boulder, CO, USA). Approximately 8 mL of triiodide solution (2 g of potassium iodide and 1.3 g of iodine dissolved in 40 mL of water with 140 mL of acetic acid) was placed in the purge vessel which plasma samples were injected. The data were analyzed using the software Origin Lab 6.1 (11).

Genomic DNA was extracted from whole blood samples according to the recommendations of the QIAamp DNA Mini Kit (catalog number 51154, Qiagen, Germany). Genotyping was performed by the Taqman system in a thermocycler (Viia7, Applied Biosystems, USA) by real-time polymerase chain reaction (PCR), thus discriminating the different genotypes for each gene. For discrimination of the *eNOS* polymorphisms -786T>C (rs 2070744) and 894G>T (rs 1799983) pre-designed genotyping assays from Applied Biosystems were used with the following catalog numbers respectively: C_15903863_10 and C_3219460_10. Genotypes for the VNTR polymorphism in intron 4 were determined by PCR and fragment separation by electrophoresis in 8% polyacrylamide gels as previously described [18–20].

Haplotypes were estimated using the PHASE program version 2.1. The possible haplotypes of the *eNOS* gene, including the three polymorphisms studied (-786T>C; intron 4b/a (VNTR) and 894G>T) were: TbG, TbT, TaG, CbG, CbT, CaG, and CaT (Table 1). Haplotypes with frequencies lower than 5% in any group were excluded from the statistical analysis.

**Table 1:** Characteristics of the participants.

General Variables	Frequency	Nitrite concentration (nM)	SBP (mmHg)	DBP (mmHg)
Gender (F/M)	358 / 71	103.29 ± 50.83 (F) 102.87 ± 45.07 (M)	122.96 ± 15.59 (F) 126.00 ±15.59 (M)	77.64 ± 10.37 (F) 79.26 ± 10.21 (M)
Age (years)	62.5 ± 8.7	-	-	-
BMI (kg/m^2^)	28.3 ± 4.8	-	-	-
***eNOS* Genotypes** (n = 429)				
g-786T>C				
TT	0.42 (179)	102.75 ± 45.83	122.95 ± 15.91	77.86 ± 9.41
TC	0.44 (191)	104.96 ± 56.64	124.95 ± 15.02	78.62 ± 11.72
CC	0.14 (59)	98.94 ± 36.93	121.20 ± 13.54	75.71 ± 7.82
4b/4a Intron 4				
bb	0.68 (290)	104.71 ± 50.69	122.92 ± 15.51	77.51 ± 10.09
ba	0.30 (128)	101.96 ± 48.16	125.34 ± 14.36	78.88 ± 10.89
aa	0.02 (11)	79.06 ± 45.47	121.72 ± 17.58	77.27 ± 10.78
894G>T				
GG	0.52 (224)	102.44 ± 48.44	124.24 ± 14.27	78.16 ± 10.73
GT	0.38 (165)	105.69 ± 55.16	123.05 ± 16.21	77.60 ± 8.84
TT	0.10 (40)	97.28 ± 31.73	122.34 ± 16.58	77.75 ± 13.64
***eNOS* haplotypes** (n = 859)				
TbG	0.51 (437)	105.69 ± 51.89	123.46 ± 14.64	77.87 ± 10.18
TbT	0.06 (55)	95.95 ±28.38	121.51 ± 21.42	79.30 ± 11.61
TaG	0.07 (57)	94.24 ± 48.14	127.14 ± 15.97	78.90 ± 9.53
CbG	0.05 (31)	89.44 ± 31.84	125.17 ± 16.81	75.95 ± 8.79
CbT	0.21 (185)	105.57 ± 53.11	123.35 ± 14.52	77.30 ± 10.18
CaG	0.10 (90)	101.72 ± 48.15	123.38 ± 14.08	78.72 ± 11.70
**Estimated Training Status (**n = 258)				
weak GFFI	0.33 (85)	96.53 ± 47.56	125.88 ± 15.54	79.23 ± 10.76
regular GFFI	0.27 (69)	100.35 ± 51.91	120.9 ± 11.42 ^a^	76.07 ± 10.11
good GFFI	0.40 (104)	112.09 ± 55.15	118.73 ± 12.02 ^a^	75.40 ± 8.74 ^a^

Note: Gender is presented in total number of participants. Age, BMI (body mass index), GFFI (general functional fitness index), nitrite concentration, SBP (systolic blood pressure and DBP (diastolic blood pressure) are presented as mean and standard deviation. The frequencies and the total number of participants are shown in % (n) respectively.

^a^ different vs weak GFFI, p<0.05.

### Training status (TS)

The "Functional Fitness Test Battery" proposed by the "American Alliance for Health, Physical Education, Recreation and Dance" (AAHPERD) was performed to estimate the TS of each participant. This battery test evaluate several capacities which involves motors tasks similar to the daily activities such as coordination, flexibility, muscular strength and endurance, dynamic agility, and cardiovascular endurance, as previously described [21, 22]. The results of each motor test were classified according to the normative values and the sun of each score were used to calculate the individual General Functional Fitness Index (GFFI) as described previously [23–25]. This procedure allowed the division of participants into TS subgroups (TS1 = weak GFFI: 0–199 points, TS2 = regular GFFI: 200–299 points, TS3 = good GFFI: 300–500 points). The original division recommended by the battery tests predicts the division of 100 in 100 points, however, due to a low frequency of participants with 0–100 and 401–500 points, and following the guidelines of Hollander and Wolf (2003), which recommended that each group should contain at least 10% of the total participants, this new division was standardized. The AAHPERD Battery Test demonstrates good reliability and criterion validity for use in older adults. The test-retest reliability coefficients for each capacity were reported in the range of r = 0.80–0.99 [22]. For this evaluation only 258 participants completed all tests.

### Statistical analysis

The distribution of genotypes and alleles for each polymorphism were evaluated by the Hardy-Weinberg equilibrium using the Chi squared test (χ^2^) (StatView, Cary, NC, USA). A priori, unusual haplotypes from the analysis (haplotype frequency less than 5%) were excluded to reduce the degrees of freedom and increase the power of the haplotype analysis. Data are reported as mean and standard deviation, with a significance level of P<0.05. The Kolmogorov-Smirnov test was used to evaluate the normal distribution of data and multiple linear regression analyses were carried out to account for factors that could influence BP and NO_2_^-^ concentrations. Age, gender, BMI, GFFI, *eNOS* genotypes and haplotypes were included as independent variables in multiple linear regression models to explain NO_2_^-^ concentrations and BP values. Data were analyzed using the SPSS 20.0 statistical package.

## Results

Table 1 shows the general characteristics of the participants. The predominance of female participants and BMI classification as overweight according to the World Health Organization [26] can be observed. There was a higher frequency of the heterozygous TC genotype of the g.-786T>C polymorphism. For the VNTR polymorphism in intron 4 a greater frequency of the ancestral genotype bb and for the 894G>T polymorphism a higher frequency of the ancestral GG genotype were also observed. Among the haplotypes, the highest frequency observed in the study was TbG (51%). Regarding the TS, the majority of participants were classified as having a good level of GFFI.

In addition, Table 1 also presented the means and standard deviations of nitrite concentrations, SBP and DBP values according to the respective groups (gender, genotypes, haplotypes and TS). Statistical significant difference was observed only when participants were divided according to TS level, which weak GFFI group showed higher values of SBP compared with regular and good GFFI groups. Similar results were observed for DBP, which showed higher values when weak GFFI and good GFFI.

The associations of *eNOS* genotype with NO_2_^-^ and BP values are presented in Table 2. It can be seen that NO_2_^-^ was influenced by GFFI and 4b/4a intron 4 independently. Interestingly, higher TS was associated with higher NO_2_^-^ and b allele in intron 4 was also associated with higher levels of NO_2_^-^. Regarding SBP and DBP, both were inversely associated with TS, as expected. Finally, it was showed a significant association of the g.-786T>C variant genotype with lower DBP. Other independent variables were not associated with changes in NO_2_^-^, SBP or DBP.

**Table 2:** Association of characterization variables, estimated training status, and *eNOS* genotypes on nitrite concentration and blood pressure values of older adults.

	Nitrite concentration (nM)		SBP (mmHg)		DBP (mmHg)	
	R^2^ = 0.09	RMSE = 50.90	R^2^ = 0.15	RMSE = 12.67	R^2^ = 0.10	RMSE = 9.66
**Source**	β	P	β	P	β	P
Age (years)	+0.26	0.533	+0.36	0.001*	-0.10	0.184
Gender (male)	+0.59	0.889	+2.41	0.023*	+1.39	0.086
BMI (kg/m^2^)	-0.62	0.439	+0.23	0.243	+0.27	0.074
GFFI	+0.08	0.016*	-0.02	0.046*	-0.01	0.001*
g.-786T>C	P = 0.586		P = 0.137		P = 0.017*	
	β	P	β	P	β	P
TT	-3.63	0.569	+2.15	0.177	+2.55	0.036*
TC	+4.34	0.360	+1.57	0.183	+1.57	0.081
CC	-0.70	0.926	-3.72	0.049	-4.13	0.004*
4b/4a Intron 4	P = 0.043*		P = 0.763		P = 0.302	
	Β	P	β	P	β	P
Bb	+19.88	0.013*	-1.10	0.579	-2.21	0.144
Ba	+5.97	0.412	-1.22	0.498	-0.27	0.847
Aa	-25.85	0.047*	+2.32	0.470	+2.48	0.313
894G>T	P = 0.156		P = 0.384		P = 0.068	
	β	P	β	P	β	P
GG	+1.50	0.819	-1.85	0.259	-2.64	0.035
GT	+10.00	0.056	-1.05	0.418	-0.97	0.326
TT	-11.50	0.173	+2.90	0.167	+3.61	0.025

Note: **SBP**: Systolic Blood Pressure. **DBP**: Diastolic Blood Pressure. **BMI:** Body Mass Index. **GFFI**: General Functional Fitness Index. **R**^**2**^: Portion of variability explained by the model. **RMSE**: Root Mean Square Error. **β:** Parameter estimate.

*Statistically significant.

Table 3 shows the association of *eNOS* haplotypes with NO_2_^-^ concentration and BP values. It can be observed that GFFI affected the NO_2_^-^concentration, SBP, and DBP independent of the haplotypes (Table 3).

**Table 3:** Association of characterization variables, estimated training status, and *eNOS* haplotypes on nitrite concentration and blood pressure values of older adults.

	Nitrite concentration (nM)		SBP (mmHg)		DBP (mmHg)	
	R² = 0.05	RMSE = 51.26	R² = 0.14	RMSE = 12.60	R² = 0.09	RMSE = 9.63
**Source**	β	P	β	P	β	P
Age (years)	+0.23	0.434	+0.35	0.001*	-0.09	0.077
Gender (male)	+0.78	0.794	+2.33	0.001*	+1.28	0.023*
BMI (kg/m^2^)	-0.55	0.321	+0.19	0.154	+0.25	0.018*
GFFI	+0.08	0.001*	-0.01	0.003*	-0.02	0.001*
Haplotype	P = 0.093		P = 0.364		P = 0.054	
	β	P	β	P	β	P
TbG	+9.37	0.029	-0.64	0.549	-0.70	0.388
TbT	+0.89	0.913	+3.63	0.071	+4.79	0.002
TaG	-7.50	0.324	+0.43	0.817	+0.05	0.972
CbG	-11.13	0.326	+0.26	0.926	-2.55	0.230
CbT	+12.20	0.017	-1.09	0.382	-1.13	0.239
CaG	-3.83	0.556	-2.58	0.106	-0.46	0.706

Note: **SBP**: Systolic Blood Pressure. **DBP**: Diastolic Blood Pressure. **BMI**: Body Mass Index. **GFFI:** General Functional Fitness Index. **R**^**2**^: Portion of variability explained by the model. **RMSE**: Root Mean Square Error. **β:** Parameter estimate.

*Statistically significant

## Discussion

The purpose of the current study was to investigate the influence of *eNOS* genotypes and haplotypes and different estimated TS levels on NO_2_^-^ concentration and BP values in older adults. According to the results it can be observed that NO_2_^-^concentration and BP values were influenced by GFFI, while the g.-786T>C polymorphism influenced only DBP and the VNTR polymorphism in intron 4 influenced the NO_2_^-^.

The data presented in Table 1 show an interesting predominance of women compared with men in the sample. Trapé et al. [27] performed a study with a similar proportion between women and men suggesting that this predominance is due to the fact that women adhere more to social, community and supervised programs of physical exercise, which could justify the lower adherence of men to health research.

The mean age of this population was 62.5 years and the BMI was 28.3 kg/m^2^. Although BMI can represent an increased risk to develop some chronic non-communicable diseases [26] and also undergoes a nutritional transition in this age group [28], participants were classified as overweight according to the Word Health Organization. So, participants were considered homogenous for BMI, avoiding the influence of obesity in the general results.

Regarding to genetic characteristics of *eNOS* polymorphisms, it was observed higher frequency of ancestral homozygous genotypes for intron 4 and 894G>T and higher frequency of the heterozygous genotype (TC) for the g.-786T>C polymorphism. Although the literature shows that the variant alleles of these genotypes are predominant in the general population, it was not observed in the participant characteristics of the current study. This result could be associated with a low risk of developing HT in the participants of this study since variant alleles have been found to be responsible for an increased risk of HT [29, 30]. Studies have shown that *eNOS* gene polymorphisms are associated with HT and some CVDs due to reduced production and/or bioavailability of NO [4, 31, 32]. However, Silva et al. [3] emphasize that even if there is a deficiency in vasodilator production of NO due to a variant allele, the maintenance of good levels of TS may be beneficial to maintain normal BP levels.

The majority of participants in the current study were classified as good level of estimated TS, which may be an important condition for the cardiovascular health of older adults (Table 1). Due to the strong relationship between estimated TS and some variables related to risk factors for CVDs, as demonstrated in some publications of our group [3, 8, 9], this result emphasizes the importance of maintaining good levels of TS to control the development of HT and CVDs in adults and the elderly [3, 33, 34].

Table 2 shows the influence of estimated TS and *eNOS* genotypes on NO_2_^-^ concentration and BP values. It can be observed that NO_2_^-^, SBP, and DBP were influenced by GFFI, regardless of genotype results. This finding is similar to a recent study by Trapé et al. [35] that demonstrated a positive effect of 12 weeks of multicomponent physical training on SBP, DBP, NO_2_^-^ concentration, redox status, and physical fitness, also regardless of genotype results. Moraes et al. [36] also performed a multicomponent physical exercise program (two sessions per week/60 minutes each session/for 12 weeks) in a Basic Health Care Unit with thirty-six hypertensive elderly people presenting a significant reduction of 6 mmHg in SBP and 2 mmHg in DBP after the intervention. An additional study by Trapé et al. [34] also showed that the practice of walking associated with another type of physical exercise (multicomponent activity) provided better results in the risk factors for CVDs and in the level of physical fitness in older adults. Therefore, these practices should be incorporated by health professionals for improvements in health and quality of life.

Regarding the influence of eNOS polymorphisms on NO2- and BP values, it was observed that the g.-786T>C polymorphism only influences DBP values. Although the literature contains some controversies about this issue, global results from the meta-analysis performed by Xie et al. [37] support that results which eNOS g.-786T>C polymorphism is significantly correlated with hypertension [37]. Moreover, the current study suggests that this polymorphism does not affect the endogenous production of NO2- which is similar to the Nagassaki study [38]. However, 42% of our participants were classified as TT for this polymorphism, which is associated with normal eNOS activity. Although CC genotype of g-786T>C presented significance effect (P = 0.049) on SBP, the relationship between them it is not possible to be confirmed since g-786T>C did not reach statistical difference (overall P-value is P = 0.137) for SBP.

Table 2 also shows that the intron 4 polymorphism exerted influence on NO_2_^-^ concentration. In the study of Marco et al. [39], the genetic polymorphism in intron 4 caused a reduction in plasma NO_2_^-^ concentration and, therefore, suggests that there may be impairment in *eNOS* protein activity, which could lead to an increase in cardiovascular risk [39]. Furthermore, in the meta-analysis of Zintzaras et al, [40], an association between hypertension and the *eNOS* intron 4 polymorphism was evidenced, especially for the b allele compared with a allele [40].

Table 3 demonstrates the effects of *eNOS* haplotypes on NO_2_^-^ and BP values. It can be observed that GFFI affected the NO_2_^-^, SBP, and DBP independent of haplotypes (Table 3). Different results were found by Nejatizadeh et al [41] who showed a significant difference in plasma NOx levels in patients with variant allele and haplotypes (g.-786T/C, 4b/4a, and 894G/T) which was significantly associated with the risk of hypertension. Moreover, the same study showed that the variants of 4b/4a and probably g.-786T/C emerged as determinants that modify the risk of hypertension, while individual polymorphisms had a marginal influence on NO_2_^-^ concentrations; however, susceptible and protective haplotypes were significantly associated with lower and higher levels of NO_2_^-^, respectively. In contrast to individual variants, the haplotypes of *eNOS* polymorphisms could represent consistent functional significance of association with phenotype (NO_2_^-^concentrations) and clinical outcome (BP) [41].

The present study did not demonstrate a significant difference in plasma NO_2_^-^concentration or BP taking into account the haplotype results (-786T/C, 4b/4a, and 894G/T). However, these differences were found in NO_2_^-^ and BP when genotype was considered. A possible explanation for this is the stratification of data into more groups and the TS evaluation. The majority of studies perform one kind of physical exercise which can promote different results. In the current study, we opted to use the TS, which represent the fitness level regardless of the physical exercise performed previously. In general, different levels of TS promote different results in these variables; however, these relationships need to be studied further.

## Conclusion

The main findings of the current study suggest that maintenance of a good level of TS can overcome the negative influence of genetic factors (intron 4) by increasing NO_2_^-^concentration and decreasing BP values.
